# Nutritional, Phytochemical, and Functional Properties of Four Edible Orchid Species from Malawi

**DOI:** 10.3390/foods13152403

**Published:** 2024-07-29

**Authors:** Felix D. Kumwenda, Jimmy Sumani, Wilson Jere, Kumbukani K. Nyirenda, Jonas Mwatseteza

**Affiliations:** 1 Department of Chemistry and Chemical Engineering, Centre for Resilient Agri-Food Systems, University of Malawi, P.O. Box 280, Zomba, Malawi; fkumwenda@luanar.ac.mw (F.D.K.); jfmwatseteza@unima.ac.mw (J.M.); 2 Department of Basic Sciences, Lilongwe University of Agriculture and Natural Resources, P.O. Box 219, Lilongwe, Malawi; 3 Department of Aquaculture and Fisheries Science, Africa Centre of Excellence in Aquaculture and Fisheries, Lilongwe University of Agriculture and Natural Resources, P.O. Box 219, Lilongwe, Malawi; wjere@luanar.ac.mw; 4 Department of Pharmacy, Kamuzu University of Health Sciences, P/B 360, Chichiri, Blantyre 3, Malawi; knyirenda@medcol.mw

**Keywords:** edible orchids, *Disa*, *Satyrium*, phytochemicals, proximate, total phenolics, functional properties

## Abstract

Terrestrial orchid tubers are an important source of food in some parts of Africa and are traditionally included in the diets of most rural communities in Malawi. However, there is limited information on the nutritional and phytochemical content of the Malawian orchids to substantiate their traditional use. The present study evaluates the nutritional and phytochemical variation of four orchid species: *Disa zombica*, *Satyrium buchananii*, *Satyrium carsonii*, and *Satyrium trinerve*, collected from the same ecological zone of Malawi. The proximate content, minerals, phytochemicals, and functional properties were analysed using official procedures. Protein ranged from 2.19% to 4.65%, whereas carbohydrate ranged from 65.24% to 80.22%, with *S. carsonii* and *S. trinerve* registering the highest protein and carbohydrate contents, respectively. Iron and potassium were highest in *D. zombica*, while sodium and calcium were highest in *S. buchananii*. The total phenolics ranged from 228.56 to 500.00 mg GAE/100 g, with *D. zombica* registering the highest. The water absorption capacity ranged from 4.10 to 10.88 g/g. Despite variable levels, the study provides evidence that orchid species contain essential nutrients and phytochemicals important for human nutrition and health. Furthermore, the functional properties can be utilised in the development of food products, such as baked products.

## 1. Introduction

Wild edible plants play a significant role in supplying food and nutritional needs, as well as increasing the health status of people in many rural parts of the world [1]. In most developing nations, numerous types of edible plants are exploited as sources of food to provide supplementary nutrition [2]. Terrestrial orchid tubers are some of the wild plants used as food in some parts of Africa, including Malawi [3].

Orchids (Orchidaceae family) are among the most widely distributed groups of flowering plants on earth and comprise about 800 genera and over 29,000 species [4]. Orchids are known for their beautiful flowers, which are diverse in shape, size, and colour, making them important ornamental plants [5]. The orchids are also used in traditional medicine in different cultures and tribes around the world. Orchids have been utilised in traditional medicine in India and China, with China alone employing approximately 300 types of orchids for this purpose [5]. In most African cultures, over 60 different orchid species are employed in traditional medicine [6].

Orchids are consumed by humans in different ways, including the globally important vanilla flavourings from vanilla spp. Seeds, salep drink from tubers, and chikanda from tubers [5]. Vanilla is mainly grown in Madagascar, where it is also used as traditional medicine, while salep is very popular in Turkey and Chikanda in Central and East Africa [7].

In several African countries, the terrestrial orchid tubers are prepared in a meat-like cake called Chinaka, or Chikanda, commonly known as African polony, which is eaten as a relish or as a snack [4]. The dish is popular in south-eastern Africa, especially Zambia, Malawi, Tanzania, and the Democratic Republic of the Congo. The dish is known by different names in different countries. It is called Chinaka, Chikande, and Kikande in Malawi, Chikanda in Zambia, and Chikanda, Kinaka, and Chikande in Tanzania, and these names also refer to the tubers themselves [4,8]. A similar dish is prepared in Cameroon, and it is called Napssié and Nyam ngub [9]. Prior to baking, the dried powder of the tubers is combined with salt and groundnut powder and then baked with either ash or sodium bicarbonate (baking soda). In Malawi, a popular method of preparing this snack omits the use of groundnut powder and uses locally prepared ash from banana leaves, dry common bean stems, leaves, or maize stalks, which is locally known as chidulo, instead of commercial baking powder [10].

The edible orchid species, with a general name of Chinaka or Chikande in Malawi, belong to the genera *Disa*, * Hebenaria*, *Satyrium*, *Brachycorythis*, and *Neobolusia*, with *Disa*, *Hebenaria*, and *Satyrium* being the preponderant species genera, and all these are harvested from the wild [11]. One study in Malawi identified 43 edible species [11].

Empirical evidence suggests that certain species are preferred over others due to factors such as flavour, baking properties, availability, and tuber size. Certain species can be prepared alone or in combination with other species, while other species, like *S. buchananii*, always require mixing with other species for preparation.

In southern Africa, research has been conducted to investigate the ethnobotany, taxonomy, conservation, and cross-border trade of edible orchids [4]. In Malawi, a review by Kasulo and others examined the distribution, diversity, and utilisation patterns of terrestrial edible orchids [12]. Until now, the variation of nutritional and phytochemical content within the orchid species indigenous to Malawi has not been reported. Additionally, to the best of our knowledge, there is no scientific report on the functional properties of the flour derived from the four Malawian species, i.e., *Disa zombica*, *Satyrium buchananii*, *Satyrium carsonii*, and *Satyrium trinerve.* Therefore, this study evaluated the nutritional, phytochemical, and functional properties of the four edible orchid species to inform their traditional use. It is expected that the findings of this study will provide impetus for potential investigations into the domestication of edible orchids to ensure improved consumption and use in the development of new food products.

## 2. Materials and Methods

### 2.1. Plant Collection and Processing

The four orchid species were collected from Luwawa Forest in Mzimba, which is located at 1590 m height with latitude 33°43′13.02″ E and longitude 12°07′17.45″ S. The plants were collected between April 2022 and August 2022 and were identified at the National Herbarium and Botanical Gardens of Malawi (NHBGM). Luwawa forms part of the northern rift valley escarpment ecological zone, with an annual rainfall range of 1020 mm to 1525 mm and mean temperatures ranging from 18.3 °C to 23.9 °C.

The tubers of orchids were transported to the University of Malawi Chemistry Laboratory for processing and analysis. The tubers were cleaned with tap water and rinsed with distilled water. The clean tubers were ground using a homogeniser and air dried for 7 days, after which they were pulverised to obtain a fine powder, which was stored in Ziploc bags pending analyses.

### 2.2. Determination of Proximate Composition

Moisture, ash, crude fat, crude fibre, and total carbohydrate contents were determined using standard methods described by the Association of Official Analytical Chemists [13].

Approximately 2.0 g of the samples in triplicate were oven-dried at 105 °C for 2 h in pre-weighed crucibles, cooled in desiccators for 30 min, and then the dried sample was weighed to a constant weight. Moisture content was obtained by expressing the loss in weight as a percentage ratio of the dry weight to the original weight of a sample.

For the ash content determination, clean platinum crucibles were incinerated in a muffle furnace (MCS-12, Biobase Biodustry (Shandong) Co., Ltd., Jinan, China), at 550 °C for 1 h, cooled for 30 min in a desiccator, and weighed on an analytical balance (Explorer, E12140, Ohaus corporation, Pine Brook, NJ, USA). To separate crucibles, 2.0 g of the powdered samples were weighed, incinerated in a muffle furnace at 550 °C for 5 h, and reweighed after cooling in a desiccator for 30 min. The ratio of the weight of ash to the original weight of the sample, expressed as a percentage, gave the ash content.

The protein percentage was determined by the macro-Kjeldahl method, which employed the following three steps: digestion, distillation, and titration. About 2.0 g of powdered sample was accurately weighed on filter paper, placed in an 800 mL Kjeldahl flask, and mixed with 30 mL of concentrated sulphuric acid and one selenium catalyst tablet. The mixture was heated using a heating mantle in a fume hood while rotating the flask occasionally until it cleared with no black specs. A filter paper without a sample was treated as a blank. The cooled digest was quantitatively filtered into a 250 mL volumetric flask and diluted to the mark with distilled water. A 5 mL aliquot of the dilute digest was put in a reaction tube in the Kjeldahl distillation unit. It was then mixed with 15 mL of 40% sodium hydroxide and steam-distilled, which released ammonia. The distillate was collected in a 250 mL Erlnmeyer flask containing 5 mL of 4% *w/v* boric acid and 5 drops of mixed indicator, and collection stopped 5 min after the distillate changed colour from red to green. The distillate was titrated against 0.01 M HCl. Crude protein was calculated using the following equation:

(1) $$Crude\;protein\;\left(\%\right)=\frac{Molarity\;of\;HCl\times 14.007\times \left(S-B\right)ml\;HCl\times 6.25\times VF}{mass\;of\;sample\;\left(g\right)\times 1000}\times 100$$

where *S* = sample titrant volume, *B* = blank titrant volume, *VF* = volume factor, 14.007 = molar mass of nitrogen, and 6.25 = conversion factor from nitrogen% to crude protein.

Crude fat was determined by the Soxhlet extraction method. About 1.0 g of sample was measured directly into a pre-weighed XT4 filter bag (ANKOLM) and sealed. The sample was dried in the oven for 3 h at 100 °C, cooled in a desiccator, and weighed. The bag was placed in the bag holder and extracted with petroleum ether (40–60 °C) in the Soxhlet apparatus, dried in the oven at 100 °C for 30 min, cooled in the desiccator, and weighed. Fat content was calculated using the following equation:

(2) $$\%Crude\;Fat=\frac{weight\;of\;pre-dried\;sample-weight\;of\;extracted\;sample}{weight\;of\;sample}\times 100$$

In determining crude fibre, about 1.0 g of samples were placed in pre-weighed bags, and the bags were securely sealed. One empty bag was included in the run. The bags were immersed in petroleum ether for 10 min to extract fat and then left to dry in the fume hood to evaporate the remaining petroleum ether. The sample-containing filter bags were subjected to boiling in a 0.225 N sulphuric acid solution for a duration of 40 min, followed by three rounds of rinsing with hot water. Subsequently, the bags were subjected to heating in 0.313 N NaOH for a duration of 40 min, followed by three rounds of rinsing with hot water. The bags were thereafter submerged in acetone and put in the fume hood to completely evaporate any remaining acetone from the sample. The bags were then dried to a constant weight in an oven at 105 °C for 2 h. The filter bags with samples were transferred to pre-weighed crucibles, incinerated in a muffle furnace at 550 °C for 2 h, and then weighed after cooling in a desiccator. The following equation was used to calculate the amount of crude fibre present:

(3) $$\%Crude\;Fibre=\frac{{(W}_{3}-(W_{1}\times C_{1}))}{W_{2}}\times 100$$

where *W*_1_ = bag tare weight, *W*_2_ = sample weight, *W*_3_ = weight of organic matter (loss of weight on ignition of bag and fibre), and *C*_1_ = ash corrected blank bag factor (running average of loss of weight on ignition of blank bag/original blank bag).

Total carbohydrate (%) was calculated by the difference as per the following equation:

(4) $$Total\;carbohydrate\;\left(\%\right)=100\%-(moisture\%+ash\%+crude\%+crude\;fibre\%+crude\;protein\%)$$

The energy content was calculated from carbohydrates (CHO), fat, and protein as outlined in the literature [14]. The mean values of crude fat, crude protein, and total carbohydrate were multiplied by 9, 4, and 4, respectively, as follows:

(5) $$Energy\;\left(kcal.\right)=9kcal./g\times g\;fat+4kcal./g\times gCHO+4kcal./g\times g\;protein$$

### 2.3. Minerals

A weighed sample was incinerated at 550 °C for 5 h and cooled. A few drops of water were added to the ash before adding 5 mL of a 1:2:3 HNO_3_/HCl/H_2_O solution. The mixture was heated gently until the brown fumes disappeared. An amount of 5 mL of distilled water was added to the residue and heated continuously until a colourless solution was obtained. The mixture was cooled to room temperature, quantitatively filtered into a 100 mL volumetric flask using Whatman No. 42 filter paper, and diluted to the mark with distilled water. This solution was run on the Atomic Absorption Spectrophotometer (AAS) (Agient model 240FS, Tokyo, Japan) to obtain values of Fe, Ca, Na, K, and Cu, while P was run on the ultraviolet–visible (UV–vis) spectrophotometer. For Ca, a 5 mL aliquot was diluted to 50 mL with a strontium chloride solution before running on AAS.

### 2.4. Total Phenolics and Total Flavonoids

The total phenolics of the extracts were determined using Folin–Ciocalteau [15] with some modifications. Firstly, 1.0 mL of extract (1 mg/mL in extraction solvent) was placed in a 100 mL volumetric flask containing 60 mL of distilled water. Then, 5 mL of a 1:10 *v*/*v* freshly prepared Folin–Ciocalteau reagent was added and mixed thoroughly. To the mixture were added 15 mL of 20% sodium carbonate, and the resultant solution was made to the mark with distilled water. The solution was incubated at room temperature for 2 h, and then absorbance was read at 765 nm on a UV–vis spectrophotometer (BK-V1000, Biobase Biodustry (Shandong) Co., Ltd., Shandong, China). A calibration curve was plotted from the absorbance values of gallic acid (10–500 mg/L) standard solutions. The concentration of the sample was reported as gallic acid equivalent (GAE) after calculating it from the following equation:

(6) $$Total\;phenolics\;\left(mg\;GAE/g\right)=\frac{R\times DF\times V}{1000\times m}$$

where *R* = the result obtained from the standard curve equation, *DF* = the dilution factor, *V* = the volume of sample stock solution, and *m* = the mass of the sample. 

The flavonoids were determined by the aluminium chloride method [16]. Briefly, 3 mL of 95% ethanol, 0.2 mL of 10% *w*/*v* aluminium chloride, 0.2 mL of 1 M potassium acetate, and 5.6 mL of distilled water were added to 1.0 mL of sample extract, and the mixture was incubated at room temperature for 30 min before reading absorbance values on a UV–vis spectrophotometer at 415 nm. A calibration curve was prepared from the absorbance of quercetin (6–100 µg/mL) standard solutions. The final concentration of the sample was determined from the following equation:

(7) $$Flavonoids\;\left(mg\;QE/g\right)=\frac{R\times DF\times V}{1000\times m}$$

where *R* is the result obtained from the calibration curve equation, *DF* is the dilution factor, *V* is the volume of sample stock solution, and m is the mass (g) of the sample.

### 2.5. Functional Properties

Bulk density was determined by a slight modification of the method reported by Melese and Keyata [17]. Briefly, 1.0 g of the orchid sample was weighed into a calibrated centrifuge tube. The tube was gently and constantly tapped against the bench until there was no further change in volume. The final volume was recorded. The following equation was used to calculate the bulk density:

(8) $$Bulk\;density\;g/mL=\frac{weight\;of\;sample}{volume\;of\;sample\;after\;tapping}$$

Water absorption capacity and oil absorption capacity were determined by a slightly modified method described in the literature [18]. Approximately 1.0 g of sample was mixed with 10 mL of water or soybean oil in a pre-weighed centrifuge tube, stirred with a stirring rod, vortexed for 2 min, and left to stand for 30 min. The mixture was centrifuged at 4000 rpm for 30 min, and the supernatant was decanted. The centrifuge tube with residue was weighed, and the water absorption capacity and oil absorption capacity (performed in triplicate) were calculated as follows:

(9) $$Water/oil\;absorption\;capacity\;\left(g/g\right)=\frac{W_{3}-W_{2}}{W_{1}}$$

where *W*_3_ is the weight of the centrifuge tube with the sample after centrifuging and decanting, *W*_2_ is the weight of the empty centrifuge tube before centrifuging, and *W*_1_ is the weight of the sample.

The solubility index and swelling power were determined by modifying the method presented by Nilusha and colleagues [19]. A 1.0 g sample was suspended in 10 mL of distilled water in a pre-weighed graduated centrifuge tube. The mixture was placed in a hot water bath at 90 °C with occasional stirring for 30 min. After cooling to room temperature, the tube was centrifuged at 3000 rpm for 30 min. The supernatant was decanted into a pre-weighed aluminium dish. The centrifuge was weighed. The aluminium dish with contents was dried in an oven at 105 °C for 2 h, cooled to room temperature, and weighed. The solubility index and swelling power were calculated using equations as follows:

(10) $$Solubilityindex\;\left(SI\right)\%=\frac{weight\;of\;supernatant}{weight\;of\;sample}\times 100$$

### 2.6. Statistical Analysis

An analysis of variance (ANOVA) was used to test if there were significant differences in nutrients, phytochemical contents, and functional properties among the four orchid species. Before conducting an ANOVA, the data were tested for normality and homoscedasticity using the Shapiro–Wilk test and the Fligner–Killeen test, respectively. The Turkey test was used to separate the means after a significant ANOVA test. All analyses were conducted at the 5% level of significance using R statistical software, version 4.3.1 [20].

## 3. Results and Discussion

### 3.1. Nutritional Content and Variation within Orchid Species

The proximate composition of the four species of edible orchids studied is presented in Table 1. The moisture content ranged between 11.84% and 15.01%. There were significant differences (*p* < 0.0001) in the mean moisture content among the four orchid species. *S. buchananii* had a significantly higher mean moisture content, followed by *S. carsonii*, *S. trinerve*, and finally *D. zombica*. Moisture content determines the stability of the food substance. A lower moisture content of a food is a good indicator of microbial stability and gives it a better shelf life for further processing [21]. The low moisture content in the studied samples explains the practice of storing dried tubers for use when not in season. The moisture content of flours below 14% can resist microbial growth, giving the flours a stable shelf life [22], and so *D. zombica*, *S. carsonii*, and *S. trinerve* can be effectively stored for a longer period for further processing with minimal risk of microbial contamination. The percentage moisture content in this study is similar to the 11.88% reported by Ola for terrestrial orchids in Nigeria [23].

The ash reflects the mineral content present in the food material, so a high ash content indicates a high mineral content [2]. In this study, there were significant differences (*p *< 0.0001) in the mean ash content among the four orchid species, with a range from 1.28% to 2.12%. (Table 1). *S. trinerve* had a significantly higher mean ash content, followed by *S. carsonii*, *D. zombica*, and finally, *S. buchananii*. The differences could be due to differences in species. The ash contents in this study were generally lower than values of 2.38–2.45% from Cameroon [9], but comparable to a study in Tanzania, which presented a range of 0.45–3.67% [24].

The protein content ranged between 2.19% and 4.65%. There were significant differences (*p* < 0.0001) in the mean protein content among the four orchid species. *S. carsonii* had a significantly higher protein content, followed by *D. zombica*, *S. buchananii*, and lastly, *S. trinerve*. There were no significant differences in the mean protein content between *D. zombica* and *S. buchananii.* Research by John Fonmboh and others presented protein contents of 1.9% and 3.5% for the two varieties Ateechteu and Lamsie of edible orchids [9], which were comparable to protein content in *D. zombica*, *S. buchananii*, and *S. trinerve* but lower than in *S. carsonii* from this study. A study on an edible orchid tuber, *Disa ukingensis*, in Tanzania presented a protein content of 5.6% [24], which was higher than the values obtained in this study. On a dry weight basis, protein content in conventional tuber crops is generally low (1–2%), and some research has presented protein contents of 1.6%, 1.4%, and 1.4% for sweet potatoes, cassava, and yams, respectively [25]. The protein values of orchid species in this study were higher than the values of conventional tubers. The contribution to protein by the orchid species as shown in this study would, therefore, not be negligible. 

The mean fat values were significantly different (*p *< 0.0001) among the four orchid species. *S. trinerve *(1.49%) had the highest fat content, and *D. zombica* (0.27%) had the lowest amount. The crude fat results in this study were comparable to values (0.995%) [26] of terrestrial orchids in India but lower than 3.96% of the wild orchids studied in Nigeria [23]. Low-fat foods contribute less to health problems related to excess fat intake, like obesity, and also enhance the storability of orchids without spoilage through rancidity [27,28]. The orchid species in the study can effectively be prepared in low-fat food formulations due to their low-fat content [19].

Crude fibre, the total amount of plant material in food, is primarily composed of cellulose, hemicellulose, lignin, and pectin, and it resists digestion by human digestive enzymes [14]. In this study, there were significant differences (*p* < 0.0001) in the mean fibre content among the four orchid species. *S. buchananii* and *S. carsonii* had significantly higher mean fibre content, followed by *D. zombica*, and lastly, *S. trinerve*. There were no significant differences in mean fibre content between *S. buchananii* and *S. carsonii*. The fibre values of *D. zombica* and S. *trinerve* were lower, while those of *S. buchananii* and *S. carsonii* were higher than the values of 4.02%, 4.95%, and 5.22% reported in the literature [9]. The differences could be due to differences in species and varieties. Crude fibre has several uses, including aiding the digestion process, preventing constipation, reducing blood sugar and cholesterol, and preventing colon cancer, among many others [29,30]. *S. buchananii* and *S. carsonii* could contribute well to the prevention of digestion-related problems, diabetes, and colon cancer.

Carbohydrates are key to the provision of energy required by the body and are essential to the structure and operation of cellular mechanisms [21]. Carbohydrates provide energy to the body when absorbed in the body [29]. There were significant differences (*p* < 0.0001) in the mean carbohydrate content among the four orchid species. The carbohydrate contents ranged between 65.24% and 80.22%. *S. trinerve* and *D. zombica* had significantly higher mean carbohydrate contents than *S. buchananii* and *S. carsonii*. There were no significant differences in mean carbohydrate content between *D. zombica* and *S. trinerve*. Similarly, there were no significant differences in mean carbohydrate content between *S. buchananii* and *S. carsonii*. The carbohydrate value obtained in this study was higher than the orchid (60.00%) in Nigeria but lower than the value (87.991%) reported for *Eulophia nuda*, an orchid in India [26]. A comparison with tuber crops shows that the orchids in the present study had lower carbohydrates than cassava flours from Sri Lanka (86.28–93.13%) [19]. However, carbohydrate contents for *D. zombica* and *S. trinerve* were within the range of 74.18–83.94% of aerial yam (*Dioscorea bulbifera*) from Nigeria [27], while the contents for *S. buchananii* and *S. carsonii* were lower. Differences in species contribute to differences in values. Generally, roots and tubers have been reported to be high in carbohydrate and are suitably served as energy staple foods [27]. This study has revealed that the orchid species found in Malawi can contribute fairly to the energy requirements of the body. 

There were significant differences (*p* < 0.0001) in the mean energy content among the four orchid species. *D. zombica* and *S. trinerve* had significantly high mean energy content, followed by *S. carsonii*, and lastly, *S. buchananii*. There were no significant differences in mean energy content between *D. zombica* and *S. trinerve*. The energy values in this study were lower than reported energy for edible orchids in India (355.51 kcal/100 g) but higher than indigenous root and tuber foods (223.37 kcal/100 g) reported in the literature [2,26]. The orchids in the study contain an appreciable amount of energy.

### 3.2. Mineral Content

The mineral analysis of the orchids showed statistically significant variations (*p* < 0.05) in the mean mineral composition of iron (Fe), copper (Cu), sodium (Na), calcium (Ca), potassium (K), and phosphorus (P) (Table 2). K was the most abundant mineral in the orchids, with a range of 98.75 mg/100 g to 169.90 mg/100 g, while Cu was the lowest, with a range of 0.49 mg/100 g to 1.56 mg/100 g. K was significantly higher in *D. zombica* and significantly lower in *S. trinerve*. There were no significant differences in K content between *S. buchananii* and *S. carsonii*. Regarding copper, *S. carsonii* had the significantly highest content, whereas there were no significant variations in copper content among *D. zombica*, *S. buchananii*, and *S. trinerve*. The Fe concentration ranged between 6.63 mg/100 g in *S. trinerve* and 16.67 mg/100 g in *D. zombica.* The Fe concentration was significantly higher in *D. zombica*, but there were no significant differences in the Fe content among *S. buchananii*, *S. carsonii*, and *S. trinerve*. *S. buchananii* had the highest quantities of Ca, followed by *S. carsonii,* and finally, *D. zombica* and *S. trinerve*. No significant differences in Ca concentrations were observed between *D. zombica* and *S. trinerve*. For P (42.07–92.20 mg/100 g), there were significant differences among the four orchid species. P was highest in *S. carsonii*, followed by *D. zombica*, then *S. buchananii*, and lastly *S. trinerve*.

Mineral micronutrients are essential for maintaining human health and enhancing protection against illnesses. They are also important constituents of muscles, tissues, nerves, teeth, bones, and blood [2]. Fe is a crucial element in the process of forming haemoglobin. Research has demonstrated that Fe, K, Mg, and Ca collaborate synergistically to effectively lower blood pressure [31]. In comparison to a review of cassava by Ferraro and others [32], the study found that *D. zombica* had a higher Fe content than cassava, ranging from 0.3 mg/100 g to 14 mg/100 g. On the other hand, *S. buchananii*, *S. carsonii*, and *S. trinerve* had Fe values that were within the range of cassava’s iron content. A comparison with the same review [32] revealed that the four orchids examined in this study had higher levels of Cu than cassava (0.2 mg/100 g–0.6 mg/100 g), but lower levels of Na (76 mg/100 g–210 mg/100 g). However, the Ca content of the orchids fell within the values observed in cassava (19 mg/100 g–176 mg/100 g).

### 3.3. Total Phenolics and Total Flavonoids

The results of the total phenolic content for the four orchid species extracted with water and methanol are presented in Table 3.

There was a significant interaction (*p* = 0.0192) between the orchid species and solvent on the mean concentration of total phenolics. The interaction with the highest total phenolics was *D. zombica* (500.00 mg GAE/100 g), while *S. carsonii* had the lowest total phenolics (228.56 mg GAE/100 g), both extracted with water. In *S. trinerve*, *S. buchananni,* and *S. carsonii*, the mean concentration of total phenolics was higher when extracted with methanol than water. A study by Do and others [33] also found that total phenolics were extracted more with methanol (40.5 mg GAE/g) than water (6.5 mg GAE/g). Possibly, there are some complex phenolics in the sample that are more soluble in methanol than water, and they possess more phenol groups or have a higher molecular weight than the phenolics in water extracts [33]. Among the three orchid species, the difference in mean total phenolic concentration when extracted using methanol and water was highest in *S. buchananni*. For *D. zombica*, the mean total phenolic concentration was higher when water was used as a solvent than methanol. A higher total phenolic content in water extracts than methanol has been reported in other plants [34]. Differences in the matrix material may also contribute to the extraction effectiveness of solvents. The total phenolics values in the study were lower than 13,900 mg GAE/100 g for edible orchids in Turkey [35] but higher than 110.7 mg GAE/100 g for Abyssinicus cultivars from Ethiopia [21]. However, the values were within the range of 140–22,900 mg GAE/100 g for wild edible flowers in Thailand [36]. Total phenolics are known for their antioxidant activities. They are also free radical scavengers, have anti-inflammatory activities, and have the ability to reduce the risk of cardiovascular diseases [36]. The high total phenolic contents in this study show that the orchids possess potential health benefits for humans.

The total flavonoid contents for the four orchid species are presented in Table 4. The results show that there were significant differences (*p*-value < 0.0001) in flavonoid content among the four orchid species when extracted with water.

*S. buchananii* had the highest flavonoid content, followed by *D. zombica*, *S. carsonii*, and lastly, *S. trinerve*. There were no significant differences in flavonoid content between *D. zombica* and *S. carsonii*. The flavonoids for *D. zombica*, *S. carsonii*, and *S. trinerve* were lower than the tubers (85.21 mg/100 g–390.65 mg/100 g) reported by other authors [25], but comparable to the tubers and roots of selected edible orchids (23.05 mg/100 g–110 mg QE/100 g) in Nepal [37]. Flavonoids are strong antioxidants, have the potential to reduce the risk of heart disease and neurodegenerative disorders, and possess anticancer properties [36]. 

### 3.4. Functional Properties

Results on the functional properties (bulk density, solubility index, swelling power, water absorption capacity, and oil absorption capacity) of the four orchid species are presented in Table 5.

The density of a food product, which determines its porosity, is a crucial characteristic that profoundly impacts the food quality, texture, and overall consumer experience [19]. The analysis of bulk density revealed statistically significant variations (*p* < 0.0001) in mean bulk densities (0.45–0.74 g/mL) across the four edible orchid species. The species *S. trinerve* exhibited the highest mean bulk density, followed by *S. buchananii, S. carsonii,* and finally, *D. zombica*. There were no significant differences in mean bulk density between *S. buchananii* and *S. carsonii*. The orchids in the study had a lower value compared to the value for potato flours (0.80–0.89 g/mL) from a study conducted in South Africa [22]. However, the orchids fell within the ranges of 0.46–0.48 mg/mL, 0.50–0.55 mg/mL, and 0.683–0.719 mg/mL, as reported in a review by Dereje and others [38] on potato flours from various locations. The investigation found that all orchids in the study had bulk densities lower than 1 mg/mL. Flours with a bulk density below 1 mg/mL are excellent for producing low-bulk weaning foods and high-energy feeds [19]. 

The water solubility index and swelling power are indications of starch hydration, and they determine the textural, pasting, and thickening properties of starch-based food preparations [19]. In this study, the water solubility index ranged between 0.38% and 1.22%, while swelling power ranged between 5.67 g/100 g and 8.71 g/100 g. There were significant differences (*p* = 0.0001) in the mean solubility index among the four orchid species. *S. carsonii* had the highest mean solubility index, followed by *D. zombica*, *S. buchananii*, and lastly, *S. trinerve*. There were no significant differences in the mean solubility index between *D. zombica* and *S. buchananii*. A comparison with other studies showed that the values of the solubility index in this study were lower than values for some cassava varieties (1.92–4.08%) studied in Sri Lanka. Swelling power measures the absorption index of the granules of the flour after heating and determines the capacity of the starch molecules to hold water via hydrogen bonding [17]. Proteins and starches are the main components that contribute to swelling [17]. There were significant differences (*p* = 0.0004) in mean swelling power among the four orchid species. There were no significant differences in mean swelling power between *D. zombica* and *S. trinerve*. There were also no significant mean swelling power differences between *S. buchananii* and *S. carsonii*. However, *D. zombica* and *S. trinerve* significantly differed from *S. buchananii* and *S. carsonii* in terms of swelling power. The swelling power values for *D. zombica* and *S. trinerve* were within the range of 7.43–10.28% from the literature [17], while those for *S. buchananii* and *S. carsonii* were lower. The variations in swelling power may be due to differences in protein content and starch composition. 

There were significant differences (*p* = 0.0033) in mean oil absorption capacity (0.48 g/g–1.22 g/g) among the four orchid species. *D. zombica* had the highest mean oil absorption capacity, followed by *S. trinerve, S. buchananii*, and finally, *S. carsonii*. There were no significant differences in the mean oil absorption capacity between *S. trinerve* and *S. buchananii*. The oil absorption capacities for *S. trinerve*, *S. buchananii*, and *S. carsonii* were lower than the oil absorption capacities for the flours (0.96 g/g–1.68 g/g) studied by Keyata and others [18], while *D. zombica* had its value within the range. All four orchids had lower values than the values (2.61 g/g and 3.21 g/g) of orchids studied in Cameroon [9].

The water absorption capacity denotes the minimum amount of water that flour can absorb under a minimum water supply [19]. The water absorption capacity of the orchids ranged between 4.10 g/g and 9.68 g/g. There were significant differences (*p* < 0.0001) in the mean water absorption capacity among the four orchid species. *S. trinerve* had the highest mean water absorption capacity, followed by *D. zombica*, *S. buchananii,* and, lastly, *S. carsonii*. The water absorption capacities of the four orchid species were higher than those of Bagana and Hambaguyta (2.09 g/g and 2.08 g/g), as investigated by Tsehay and others [21]. Nevertheless, the values were similar to the values (7.29 g/g and 9.10 g/g) published in the literature for orchid tubers in Cameroon [9]. Ngoma and colleagues [22] found that a high water absorption capacity is indicative of the flour’s efficacy in the manufacturing of various food items, such as bakery products and dough. The high water absorption capacity observed in this study suggests that the four edible orchids could be suitable for use in bakery items.

## 4. Conclusions

The results from the study have revealed that the four orchid species, namely *D. zombica*, *S. buchananii*, *S. carsonii*, and *S. trinerve,* contain essential nutrients and phytochemicals in substantial amounts, which are important for improving human nutrition and health. The contents of protein and carbohydrates ranged from 2.19 to 4.65% and from 65.24 to 80.22%, respectively. A considerable amount of total phenolic content (ranging from 228.56 to 500.00 mg GAE/100 g) was obtained. The results demonstrated that the four orchid species exhibited desirable functional properties that could be explored for the development of various food products. Furthermore, significant differences in nutritional content, phytochemical content, and functional properties were observed in the four orchid species. Evidently, the consumption of orchids and their use in food product development could be recommended to improve human nutrition and health, as well as the development of food products for household consumption and sale as an income-generating activity.

## Figures and Tables

**Table 1:** Proximate composition of orchid species.

Orchid	Moisture (%)	Ash (%)	Protein (%)	Fat (%)	Fibre (%)	Carbohydrate (%)	Energy (kcal/100 g)
*Disa zombica*	11.84 ± 0.04 ^d^	1.75 ± 0.02 ^c^	3.28 ± 0.00 ^b^	0.27 ± 0.03 ^d^	3.33 ± 0.03 ^b^	79.50 ± 0.11 ^a^	331.13 ± 0.43 ^a^
*Satyrium trinerve*	12.30 ± 0.09 ^c^	2.12 ± 0.01 ^a^	2.19 ± 0.00 ^c^	1.49 ± 0.06 ^a^	1.74 ± 0.01 ^c^	80.22 ± 0.03 ^a^	329.65 ± 0.12 ^a^
*Satyrium buchananii*	15.01 ± 0.08 ^a^	1.28 ± 0.01 ^d^	3.28 ± 0.00 ^b^	1.02 ± 0.05 ^b^	14.06 ± 0.01 ^a^	65.42 ± 0.12 ^b^	274.81 ± 0.47 ^c^
*Satyrium carsonii*	13.73 ± 0.11 ^b^	1.81 ± 0.01 ^b^	4.65± 0.16 ^a^	0.54 ± 0.04 ^c^	14.00 ± 0.39 ^a^	65.24 ± 0.40 ^b^	279.55 ± 0.95 ^b^
*p*-value	<0.0001	<0.0001	<0.0001	<0.0001	<0.0001	<0.0001	<0.0001

Note: Means having the same superscript in any given column are not significantly different at the 5% level of significance.

**Table 2:** Mineral nutrient content of four orchid species (mg/100 g).

Orchid Species	Fe	Cu	Na	Ca	K	P
*Disa zombica*	16.67 ± 0.96 ^a^	0.49 ± 0.12 ^b^	15.50 ± 0.77	36.66 ± 1.27 ^c^	169.90 ± 17.64 ^a^	72.13 ± 3.59 ^b^
*Satyrium buchananii*	7.94 ± 2.41 ^b^	0.62 ± 0.22 ^b^	16.98 ± 0.51	93.14 ± 9.58 ^a^	107.52 ± 11.55 ^ab^	55.70 ± 0.28 ^c^
*Satyrium carsonii*	8.66 ± 0.31 ^b^	1.56 ± 0.03 ^a^	16.84 ± 0.23	64.50 ± 0.50 ^b^	167.40 ± 18.75 ^ab^	92.20 ± 3.64 ^a^
*Satyrium trinerve*	6.63 ± 0.71 ^b^	0.89 ± 0.07 ^b^	14.94 ± 0.11	18.76 ± 2.16 ^c^	98.75 ± 13.27 ^b^	42.07 ± 3.09 ^d^
*p*-value	0.0031	0.0016	0.0413	<0.0001	0.0196	<0.0001

Note: Means having the same superscript in any given column are not significantly different at the 5% level of significance.

**Table 3:** Mean concentration of total phenolics for four orchid species using water and methanol solvents.

Orchid Species	Solvent	Total Phenolic Concentration (mg GAE/100 g)
*Disa zombica*	Water	500.00 ± 35.95 ^a^
	Methanol	309.52 ± 53.02 ^ab^
*Satyrium trinerve*	Water	414.29 ± 28.57 ^ab^
	Methanol	452.38 ± 17.17 ^ab^
*Satyrium buchananii*	Water	252.19 ± 71.26 ^ab^
	Methanol	447.62 ± 81.37 ^ab^
*Satyrium carsonii*	Water	228.56 ± 57.74 ^b^
	Methanol	295.24 ± 56.14 ^ab^
	*p*-value = 0.0192	

Note: Means having the same superscript in the column are not significantly different at the 5% level of significance.

**Table 4:** Flavonoid content in four orchid species extracted with water.

Orchid Species	Flavonoid Content (mg QE/100 g)
*Disa zombica*	72.8788 ± 7.47 ^b^
*Satyrium trinerve*	31.4646 ± 1.34 ^c^
*Satyrium buchananii*	91.5657 ± 1.01 ^a^
*Satyrium carsonii*	68.8384 ± 2.67 ^b^
*p*-value < 0.0001	

Note: Means having the same superscript in any given column are not significantly different at the 5% level of significance.

**Table 5:** Mean functional properties of the four orchid species.

Orchid	Bulk Density (g/mL)	Solubility Index (%)	Swelling Power (g/100 g)	Oil Absorption Capacity (g/g)	Water Absorption Capacity (g/g)
*Disa zombica*	0.45 ± 0.03 ^c^	0.71 ± 0.04 ^b^	8.50 ± 0.48 ^a^	1.22 ± 0.04 ^a^	9.68 ± 0.15 ^b^
*Satyrium trinerve*	0.74 ± 0.02 ^a^	0.38 ± 0.03 ^c^	8.71 ± 0.42 ^a^	0.53 ± 0.02 ^b^	10.89 ± 0.11 ^a^
*Satyrium buchananii*	0.63 ± 0.002 ^b^	0.69 ± 0.12 ^b^	6.67 ± 0.05 ^b^	0.48 ± 0.20 ^b^	5.11 ± 0.10 ^c^
*Satyrium carsonii*	0.63 ± 0.001 ^b^	1.22 ± 0.02 ^a^	5.67 ± 0.02 ^b^	0.58 ± 0.004 ^b^	4.10 ± 0.07 ^d^
*p*-value	<0.0001	0.0001	0.0004	0.0033	<0.0001

Note: Means having the same superscript in any given column are not significantly different at the 5% level of significance.

## Data Availability

The original contributions presented in the study are included in the article, further inquiries can be directed to the corresponding author.
